# All together now – patient engagement, patient empowerment, and associated terms in personal healthcare

**DOI:** 10.1186/s12913-022-08501-5

**Published:** 2022-09-02

**Authors:** Emily Hickmann, Peggy Richter, Hannes Schlieter

**Affiliations:** Faculty of Business and Economics, Research Group Digital Health, Technische Universität Dresden, Helmholtzstr. 10, 01069 Dresden, Germany

**Keywords:** Concept map, Conceptualisation, Patient-centredness, Patient empowerment, Patient engagement, Systematic literature review

## Abstract

**Background:**

Patients as active partners in their personal healthcare are key drivers to reducing costs, securing an effective usage of resources, and ensuring patient-provider satisfaction. Even though these benefits are acknowledged, a theoretical framework for the plethora of concepts used in this context, such as patient engagement, patient empowerment, or patient involvement is missing. Furthermore, the heterogeneous or synonymous usage of these terms leads to miscommunication, missing standard conceptual measures, and a deficiency in theory building and testing. Our objective is to show what the relationships and distinctions between concepts focussing on patients as active partners in their personal healthcare are.

**Methods:**

A systematic literature review was conducted to consolidate terms related to patients’ having an active role in their healthcare. From 442 articles screened in PubMed, a final set of 17 papers was included. Any articles conceptualising or presenting relationships between the concepts were included. Information was synthesised, and contradictions were unravelled systematically. The concepts and their relationships are structured and represented by employing a concept map.

**Results:**

Patient-centredness is a concept dominantly influenced by health care providers and can enhance patients’ competencies, attitudes, and behaviours towards their personal healthcare. Enabling patients to become more empowered can ultimately lead to their greater involvement and engagement. Fostering an active role of patients can also increase their adherence to the care pathway. In general, patient engagement seems to be the most conclusive and furthest developed concept in terms of turning patients into active partners in their personal healthcare.

**Conclusions:**

We plead for a stricter demarcation and therefore a terminological standardisation of the terms in the future to avoid further ambiguity and miscommunication. The concept map presents a basis for a uniform understanding and application of the concepts. Through a comprehensive understanding of the terms and their dimensions, relationships between the concepts can be utilised, measures can be derived, and theory building and testing can be enhanced, leading to better acceptance and utilisation of concepts in healthcare services. Furthermore, patient engagement is presented to be the most conclusive and furthest developed concept in the subject area.

**Supplementary Information:**

The online version contains supplementary material available at 10.1186/s12913-022-08501-5.

## Background

Due to current healthcare challenges, such as an ageing population, an increased number of patients with multimorbidity and chronic diseases, as well as regional care deficits healthcare services are permanently under the pressure to accomplish a higher workload in a time-restricted and effective manner. Enabling patients to become co-managers of their care processes is a key component in creating a high-performing and cost-efficient healthcare system [1]. Positive effects include a more effective and appropriate resource allocation, increased patient and provider satisfaction, increased usage of preventive services, and improved health outcomes [2, 3]. Despite the existing evidence and the need for patients to become active partners in their healthcare, the conceptualisation, delimitation, and relationships of related terms, including patient engagement, patient empowerment, patient involvement, or patient-centred care are fragmented, if not even missing. This results in

- an inconsistent usage of terms [4, 5],
- a poorer understanding and communication about the topic amongst researchers, patients, healthcare providers, and policymakers [4, 6],
- a lack of standard conceptual measures and evaluation tools, leading to limited comparability of studies, interventions, and policies [5, 7],
- a deficiency in theory building and testing, for instance, to understand which concepts or combination of concepts leads to the best results.

Accordingly, this paper aims to answer the research question: What are the relationships and distinctions between concepts focussing on patients as active partners in their personal healthcare? Hereby, the paper has an exclusive focus on the micro level of these concepts, i.e., patients in relation to their well-being, and not the inclusion of patients at an institutional level or in healthcare research and policymaking. Furthermore, only research that specifically targets definitions, conceptualisations, relationships, and distinctions between the terms will be considered. The remainder of this article is structured as follows: The method of creating a concept map, including the performance of a systematic literature review, is described. Results are given by describing the individual terms, their relationships, and the depiction and explanation of the concept map. The paper closes with a discussion of future research opportunities.

## Methods

**Fig. 1:**
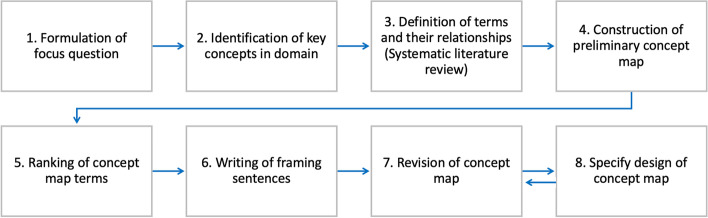
Method of concept map creation based on Novak and Canas [8] and Dubberly [9]

As we are aiming to create a concept map consolidating all terms addressing the active role of patients in their healthcare, the method is based on the steps to create a concept map proposed by Novak and Canas [8] and Dubberly [9] and is presented in Fig. 1. Novak and Canas [8] describe concept maps as a *„graphical tool for organizing and representing relationships between concepts indicated by a connecting line linking two concepts*.“

The focus question of the concept map (1) is identical to the research question of this paper: What are the relationships and distinctions between concepts focussing on patients as active partners in their personal healthcare? To identify the relevant concepts (2), a brief search was carried out in Google, Google Scholar, and PubMed using the following terms as a starting point: patient engagement, patient empowerment, patient involvement, shared decision-making, and patient-centred care. Further related terms identified through the search are person-centred care, person-directed care, self-care, self-management, health literacy, patient enablement, patient activation, patient compliance, and patient adherence.

### Systematic literature review

Step three of the method encompasses the definition of terms and their relationships. For this purpose, a systematic literature review, following the guidelines proposed by Rowley and Slack [10] and using the PRISMA checklist for guidance, was performed in PubMed. The information obtained in the brief search was used to create the following search string.

((Patient) AND (empower* OR activat* OR engage* OR enabl* OR involve OR participation OR centred* OR orientation OR self-management OR self-care OR shared decision making OR adherence OR compliance)) AND (ontology OR definition* OR concept* OR terminolog* OR relation* OR taxonomy)

**Fig. 2:**
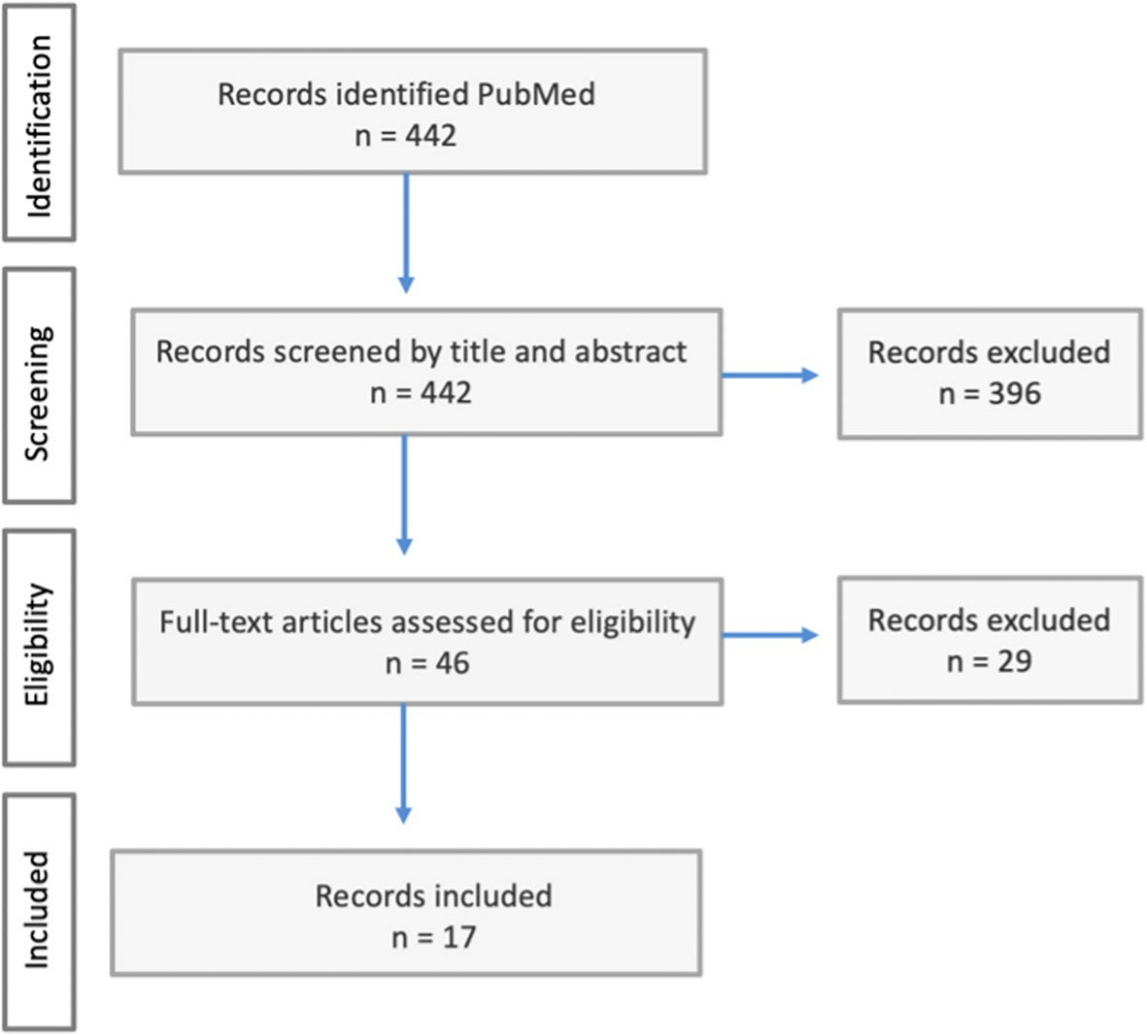
PRISMA Flow Diagram of literature search and selection process

The literature selection process is depicted in Fig. 2. The search resulted in 442 articles. First, titles and abstracts were screened, and corresponding to the inclusion and exclusion criteria named below, 396 articles were excluded. Second, the full text of the remaining articles was assessed for eligibility, and a final set of 17 papers (see Additional file 1) was included in the study. These 17 papers originate from 12 different countries and most often utilised the method of a concept analysis or a literature review. Articles were included if two main premises were fulfilled: i) the research paper had the main focus on conceptualising at least one of the included concepts, either through definition, descriptive attributes, relationships, or distinctions, and ii) the concepts were used in a micro-level context, so in relation to the patient’s personal healthcare.

Due to this very narrow set of inclusion criteria, we restricted the PubMed database search to the search field of titles. It was assumed that papers mainly focusing on conceptualisation would include one of the alternative terms (ontology, definition, and so on) in their title. This assumption was confirmed in a pre-test with 50 articles. Furthermore, we only searched for articles in English or German language. No further filters, for example concerning the date of publication, were used. It can, however, be noted that PubMed tracks articles back to 1966 [11].

### Concept map creation

The information retrieved from the 17 articles was used to create a preliminary concept map draft (4). Therefore, contradicting statements found in the literature review needed to be analysed and unravelled. The most i) common and ii) rigorously scientifically proven argumentative strings were used for the concept map creation. For example, a contradiction found in the literature review concerned the concepts of patient involvement and patient participation. Higgins et al. [12] state that both concepts focus exclusively on the patient rather than the relationship and interaction with the healthcare provider. In contrast, three other sources reasoned that patient participation is dependent on the existence of a relationship with the healthcare provider [6, 13, 14]. The second conclusion was used for concept map creation not only because more studies agreed with it but also because, in contrast to Higgins et al. [12], two of the three studies supporting this conclusion primarily focused on patient participation and, therefore, arguably, gained a deeper insight into the concept. All contradictions found during the literature review and individual choices made to unravel them are listed in Additional file 2.

Next, terms were ranked (5) according to common or distinguishing characteristics in the literature review, such as the relationship to the healthcare provider. According to the relationships between the concepts, framing words were added to the arrows (6). The revision of the concept map (7) followed an iterative process, including peer reviews. These occurred in form of two successive focus groups with six fellow researchers in the domain of digital health and information system research, who are experienced with theoretical and practical issues of enhancing patient empowerment and engagement. The discourse in these focus groups also had a large impact on concept map design and representation (8).

## Results

The following section explains the concepts individually and in relation to one another. The results are aggregated in a concept map, which illustrates the distinctions and relations of the described concepts.

### Patient empowerment

**Fig. 3:**
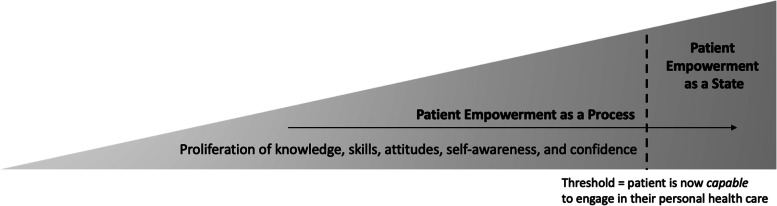
Patient empowerment as a process and as a state

The concept of patient empowerment can be traced back to philosophers such as Hegel or Sartre and critical social theory. For example, black power, women’s liberation, or gay rights are linked to the empowerment concept [15]. Within the healthcare field, patient empowerment relates to a patient’s proliferation of knowledge, skills, attitudes, or self-awareness, combined with the confidence to participate in their care [12]. Comparable to many political incentives, patient empowerment aspires to increase power for a specific group of people (in this case, patients). A comprehensive review by Cerezo et al. [5] concludes that patient empowerment is *“an enabling process or an outcome of a process involving a shift in the balance of power”* [5]. As can already be perceived in this definition, two different implications of patient empowerment can frequently be found in the literature. The first describes patient empowerment as a process (i), which enhances the patient’s capacity to think critically and make autonomous, informed decisions. The second depicts patient empowerment as a patient’s state of being empowered (ii). For example, a patient acquiring new knowledge through online reading is in the process of patient empowerment (i). The focus here shifts to activities and inputs that increase the patient’s ability and motivation. As a state (ii), patients are empowered if they feel confident enough to participate actively in consultations or self-management. The emphasis here lies on “feels confident enough,” meaning that no behavioural change must occur (i.e., the patient only needs the attitude that he or she can engage in their healthcare). It must be noted that even when a patient feels confident enough to participate, the process of empowerment will still be enhanced further through the engagement process itself (see Fig. 3).

In accordance with French and Raven’s [16] classic typology of power, patient empowerment complies with both legitimate power – derived from the position as a patient in a patient-centred healthcare environment—and expert power – derived from an individual’s expertise by learning and experience. This leads to the question, if empowerment can be taken by a patient or if it must be given to them. We argue that the digitalisation and the resources made available through the world wide web increasingly support patients in being able to “take” the power they need, without necessarily being reliant on the relationship with a provider. Healthcare providers and a patient-centred healthcare environment, however, have the capability to enhance this process, for example by providing understandable and well-structured information.

### Patient activation

Patient activation is often used synonymously to patient empowerment. For example, Higgins et al. [12] state: *“Activation is patient-focused and indicates the patient’s confidence and skill to engage in care*.” This description would also be accurate for patient empowerment, revealing a prominent conceptual overlap, as both refer to a patient’s attitude of being able to engage in their healthcare. To explain the differences between empowerment and activation, the review by Fumagalli et al. [6] concentrated on comparing measurement scales for both concepts. While a measure for empowerment would contain generalised items such as “Do you feel powerful most of the time?”, an activation measure would concentrate on specific domain knowledge, such as “Do you know why you are supposed to take this medication?”. It was concluded that activation focuses more precisely on specific improvement goals for diseases. Empowerment is broader, giving patients the capacity to make decisions in a broader context (i.e., their health) [6].

### Patient enablement

Patient enablement focuses on the general acquisition of skills and knowledge to engage in healthcare. This can be achieved through the patient-provider relationship or personal efforts. These could, for example, include exchanges with other patients, participation in health education programs, or seeking relevant information online. This makes patient enablement and patient empowerment two very similar concepts. However, there is a clear distinction explaining why they cannot be used as synonyms. Patient empowerment encompasses the idea that the patient must have gained power through acquiring knowledge and skills. Patient enablement only focuses on procuring these assets, meaning that enablement is a preliminary competence, which can potentially progress into patient empowerment. This can also be perceived in Castro et al. [4], where next to the *“enabling process,”* additionally *“personal change”* and *“self-determination”* are described as attributes of empowerment [6]. Health literacy, as a personal asset: *“a set of personal, transferable skills that can be developed to support greater independence in health decision-making through a structured exposure to targeted and personalized information”* can be categorised as a part of patient enablement [17].

### Patient engagement

Patient engagement is, compared to the other concepts, relatively new in the literature debate and arguably the one with the most conclusive breadth of concept [18]. Multiple different manifestations can be found in current literature. A comprehensive concept analysis was performed by Higgins et al. [12], including 96 articles that utilised the concept of patient engagement. Four main attributes were identified: i) personalisation of interventions or strategies according to the individual needs of the patient, ii) ability and confidence of patients to obtain necessary resources, iii) commitment (willingness) of the patient, and iv) therapeutic alliance. This last attribute is substantial for the differentiation to other terms, as it means that a sustained connection to a healthcare provider is always a component of patient engagement. It can be summarised that for a patient to engage, they must be empowered, as they need motivation and the ability to participate in care. Additionally, the healthcare provider must maximise the potential and the opportunities for patients to engage. This could, for example, be done by facilitating access to resources, personalising the care plan, or creating a mutual and trustworthy relationship. Both patient and provider aim to reach a shared healthcare goal [18].

### Patient-centred care, person-centred care, person-directed care

The concept of patient-centred care was introduced in 1969 as a different way of medical thinking. The physician’s perspective should be broadened to incorporate the human being behind the patient. Therefore, the individuals’ reasoning, will, feelings, and needs should be considered [15]. The Institute of Medicine defines patient-centred care as *“care that is respectful of and responsive to the preferences, needs, and values of the individual and ensuring that the care recipient’s values guide all clinical decisions”* [19]*.* A content analysis by Scholl et al. [20], covering 417 articles, found 15 dimensions of patient-centred care. A dimension that seems to be unique to patient-centredness is the characteristic of the clinician*,* which is not only described as a set of attitudes towards the patient but also as self-reflectiveness and medical competence. Even though dimensions, such as the patient-clinician relationship, patient-clinician communication, or involving the patient in care are present, it is noticeable that all aspects of patient-centred care seem to be initiated by the physician (i.e., describing sentences often start with *“the physician aims…”*). This is an essential difference from other related concepts, for example, patient participation, where the main aspects are portrayed from the patient’s perspective. Describing sentences are generally formed in the pattern of *“the patient is willing to…”* or *“the patient decides…”* [5].

Holmström and Röing [15] compared the concepts of patient-centredness and patient empowerment, concluding that patient-centredness is of great value for patient empowerment. Two aspects should be considered in the relationship between patient-centred care and patient empowerment. First, patients can empower themselves, meaning that patient-centredness can enhance patient empowerment, but is not a strictly necessary antecedent [15]. Second, patient-centred care can help a physician identify if a patient does not want to be empowered. Therefore, not supporting the empowerment of a patient can still be patient-centred care. Patient-centred care also enhances patient engagement, as the personalisation of the care process is an essential aspect of engagement [12]. Furthermore, research has shown that patient-centred interactions promote adherence [21].

The concept of patient-centred care is closely connected to person-centred care. The International College of Person-centred Medicine describes person-centred care as a *“medicine of the person, for the person, by the person, and with the person”* [20]*.* Kumar and Chattu [19] argue that the main difference between these terms is that person-centred care focuses on the whole person, while patient-centred care only focuses on the person as a patient. Person-centred care would, therefore, include the entire topic of prevention. However, Scholl et al. [20] conclude that the concepts are identical. Person-directed care is also a term related to the two already described concepts. Kumar and Chattu (2018) conclude that person-directed care focuses more on putting individuals in control of decisions about their care. It can be assumed that the three concepts of patient-centred care, person-centred care, and person-directed care overlap thematically, even if it is not entirely clear if they can be used as synonyms.

### Patient participation and patient involvement

Two concepts that directly relate to the behaviours of patients are participation and involvement [4]. An extensive literature review on patient participation in nursing care was performed by Sahlsten et al. [14]. Attributes, antecedents, and consequences of the concept are portrayed. *“The patient obtains sufficient, appropriate, understandable, and meaningful information and knowledge in order to feel confident”* is an antecedent of patient participation given in the study. Gaining knowledge, skills, and confidence is patient empowerment and, therefore, an antecedent of participatory behaviour. Interestingly, patient empowerment (as well as decreased vulnerability) is also listed as a consequence of participation, meaning that the process of empowerment is further enhanced through patient participation.

When now considering patient involvement, it becomes clear why shared decision-making is an example of patient participation and why self-care and self-management are examples of patient involvement. Several sources agree that patient participation depends on an established relationship with the healthcare provider, meaning that autonomous actions or decisions by the patient are not forms of patient participation [4, 6, 13, 14]. In contrast, patient involvement is not necessarily in cooperation with the healthcare provider.

When reflecting on patient engagement in relation to patient participation, it becomes clear that parts of engagement encompass patient participation [12]. A central aspect of patient engagement is the therapeutic alliance, which is the realm of patient participation.

### Self-management, self-care, and shared decision-making

Involving or participatory actions include self-management, self-care, and shared decision-making. These concepts are explained briefly to contribute a complete overview of the topic. Self-management in healthcare refers to an *“individuals’ ability to manage the symptoms, treatment, physical and psychological consequences and the lifestyle changes inherent in living with a chronic condition”* [22]. Self-care thematically overlaps with self-management. However, self-care is the broader concept and refers to individual responsibilities for healthy lifestyle behaviours, such as maintaining good psychological health, meeting social needs, caring for minor ailments and long-term conditions, using services effectively, or maintaining health after acute illness [22, 23]. Shared decision-making implies an active engagement of patients and providers in the decision-making process by sharing information and personal values. Components include the definition of the problem that needs to be addressed, a presentation of the available options, and a discussion between the patient and the professional care provider on the advantages and disadvantages of each option [24, 25]. Again, to self-manage, self-care, or decide, knowledge and skills must be acquired first (i.e., patient enablement and empowerment). For example, a diabetes patient can only self-administer the correct insulin dosage if they know about the disease and insulin’s effect on their body. Additionally, the patient needs the skill to inject the medication independently.

### Patient adherence and compliance

Patient adherence and compliance are, in contrast to the other explained terms, already used very commonly. They refer to how patients’ behaviour corresponds to recommendations from the healthcare provider [26]. There is an essential distinction between the terms. Compliant patients accept and follow the physician’s recommendations due to their higher hierarchical status. In contrast, adherence implies an active role of the patient and, therefore, a self-motivated decision to adhere to the jointly developed recommendations. Due to its emphasis on agreement, the concept of adherence supersedes patient compliance [27]. For example, a study by Deniz et al. [28], using a structural equation model to synthesise the results of a survey with 399 participants, concluded that shared decision-making has a significant and positive influence on patient adherence. It can therefore be argued that adherence and compliance are an outcome of enabling patients to become active partners in their healthcare.

### Concept map

**Fig. 4:**
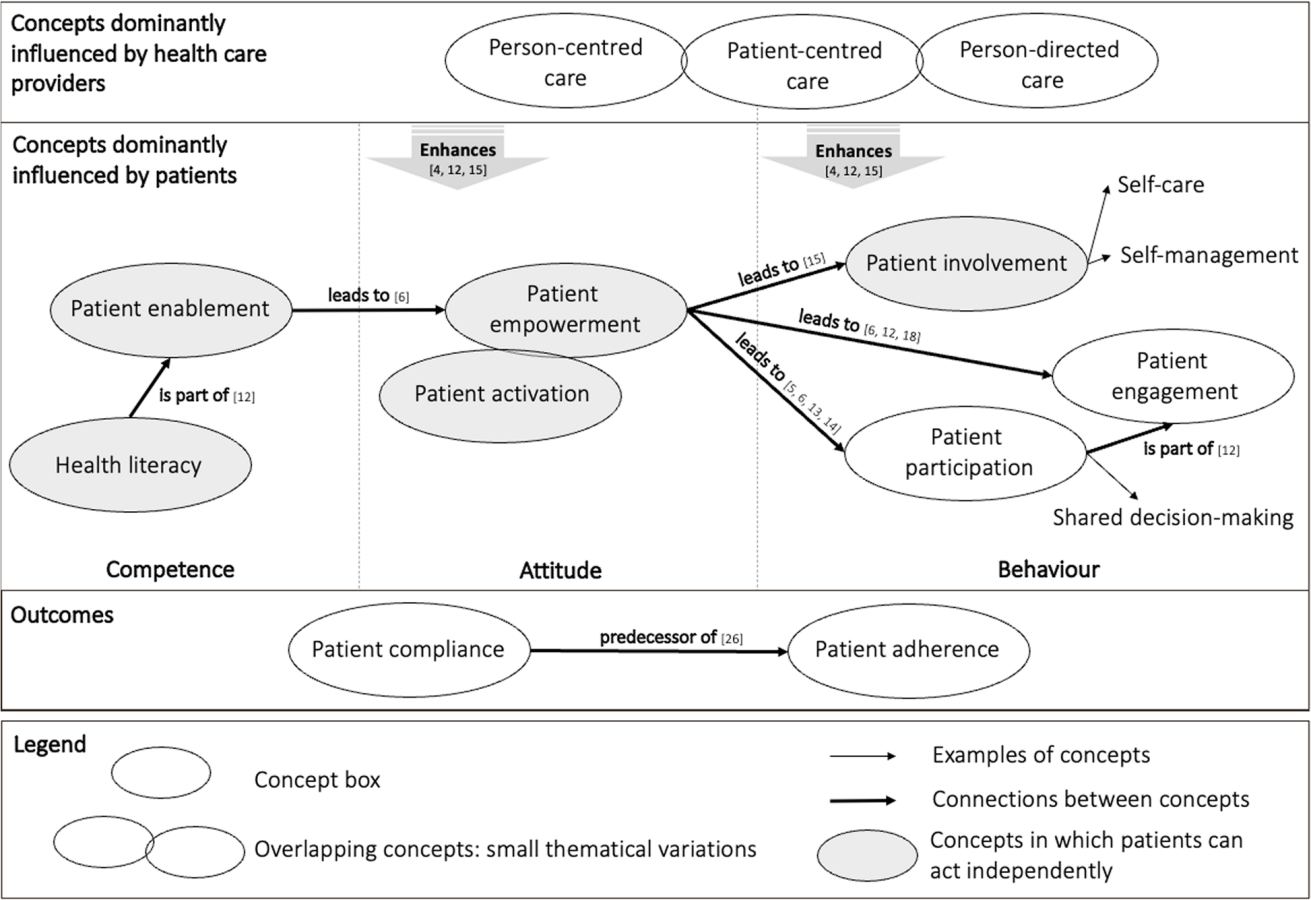
Concept map: relationships and distinctions between concepts focussing on patients as active partners in their healthcare

Figure 4 summarises the central relationships and distinctions between the analysed concepts. Concepts with minor thematical variations, sometimes also used as synonyms, are represented by overlapping circles. The concepts are divided into three main groups displayed in temporal order from left to right: competencies, attitudes, and behaviours. The elements to the left of a concept are the concepts antecedents, and the concepts to the right are the consequences. Competences describe patients’ proliferation of skills and knowledge. Attitudes describe patients feeling of power that makes them capable of a certain behaviour, such as patient engagement. A behaviour then describes patients actively doing something, for example, self-management. These consequences can but must not occur as, for example, a patient could always decide not to be engaged, even though he or she is empowered.

A distinction of the concepts that can be derived from the map is if a patient has the possibility to act independently, meaning that they are not reliant on the relationship with a healthcare provider to be, for instance, enabled, empowered, or involved. Still, a patient-centred (or person-centred or person-directed) approach can have a beneficial influence on the concepts and therefore enhance the patient’s process. In distinction to the grey-coloured concepts, the definition of patient participation and engagement already encompasses the relationship with a healthcare provider. The same is true for patient compliance and adherence, which can be classified as outcomes of enabling patients to become co-managers in their healthcare. It must be noted that these two outcomes are not a conclusive list of consequences, they are represented on this map due to their thematical proximity to enabling patients to become active partners in their healthcare.

Concepts can be divided into concepts dominantly influenced by the physician and concepts mainly influenced by patients. Attributes related to patient-centred care are generally described from the physician’s perspective and are initiated from their instance (for example “the physician aims to….”). In contrast, concepts dominantly influenced by patients, are portrayed from a patient’s perspective (for example “the patient is willing to…”).

Several concepts have recursive relationships to the concepts before them. This means that one concept leads to another but can simultaneously strengthen the concept it originates from. For example, the empowerment process will also be enhanced further by patient engagement. To simplify the concept map, only the general trends are depicted, and positive influences on prior concepts were only described in the explanations above.

## Discussion

The concept map, depicting relationships and distinctions between terms related to patient empowerment and patient engagement, holds implications and potential for research, healthcare services, and evaluation. These implications and the limitations of the study will be discussed in the following section.

### Implications

In terms of future research, the concept map provides an extensive overview of concepts relating to the active role of patients in their healthcare. Arguably, this overview and differentiation of the individual concepts in the domain of patient engagement are prerequisites for eliciting a single concept in-depth—especially as terms have been used interchangeably in literature so far. The concept map also serves as a rigorously derived foundation for theory building and testing, as hypotheses can directly be derived from the described conceptual relations. The map illustrates which concepts could function as levers to others, for example, an intervention aiming at patient empowerment would have to focus on improving patient enablement and health literacy, as well as a patient-centred approach to enhance the empowerment process of patients. Such assumptions may support the development and testing of patient engagement theories. Another aspect, especially for researchers, is that the concept map enhances understanding and creates a common communication basis. For example, in scientific articles, a large proportion of the introduction or the state-of-the-art section is often dedicated to creating terminological clarity before the actual findings can be presented. When researchers instead refer to the proposed concept map, this process is eased and a terminological basis for the article is established.

The concept of patient engagement appears to be the most conclusive and furthest developed concept. As the concept map shows, several other concepts lead to or are a part of patient engagement. Therefore, it could be argued that especially patient engagement is a promising focal point for future research. When considering this in the light of existing research, the theory of planned behaviour [29] is an interesting focal point to discuss how the patient’s beliefs, presumably partially formed in relation to the preceding concepts in the concept map, link to the behaviour of patient engagement. The theory describes three main dimensions, that shape an individual’s intention to, in our example, engage in their healthcare: i) the attitude towards a behaviour, ii) the subjective norm and iii) the perceived behavioural control. Arguably, when considering the concept map, especially the perceived behavioural control (iii) relates to many of the concepts leading into patient engagement. Perceived behavioural control is to some extent dictated by the available resources and opportunities [29]. Obtaining the necessary resources is the process of patient enablement and patient empowerment [5] while creating opportunities for engagement is an integral part of a patient centred approach [30]. Also, the subjective norm (ii), relating to beliefs about what others may think of patients engaging in their healthcare, can be influenced by the concept of patient-centred care. Arguably, the opinion of the healthcare provider is a decisive factor, so if in the opinion of the patient the provider will approve of the patient engaging in their care. Therefore, the shift (especially in the minds of physicians) from the paternalistic care model to a patient-centred one, based on an equal and trusting partnership is crucial [31]. Finally, considering the attitude towards a behaviour (i), the patient must believe that engaging in their healthcare will have a positive influence on their health status or their life in general. This could, for example, occur through a proliferated feeling of control that can be gained through the empowerment process. Furthermore, the patient’s attitude towards engagement may also be influenced by his or her belief that this behaviour may have a positive influence on the relationship with the healthcare provider. It must, however, be noted that the discussed concepts only cover aspects of what the dimensions of the theory of planned behaviour may be influenced by, and further research on what other aspects could undermine these beliefs is necessary. Nonetheless these considerations show that the temporal order used in the concept map can at least partially be reflected in the light of an existing theory.

Engaging patients has become a cornerstone for a high-quality healthcare service provision [32]. When considering dimensions or tools to support an active role of patients, either independently through the patient or in relation to the service provider, the respective concepts of interest can be derived from the concept map. This is relevant to a variety of stakeholders providing healthcare services, who can apply the results of the concept map depending on the setting in which a concept shall be introduced. For example, developers of a health promotion app, independent of a specific disease, would primarily focus on the dimensions and implementation possibilities of concepts in which the individual is not reliant on a provider relationship. In contrast, clinicians striving to activate patients or improve patient-provider communication would concentrate on the concepts involving patients’ relationships with care providers. The concept map can be used as guidance to describe practical patient engagement application guidelines for the main user groups, i.e., policymakers, healthcare service providers, healthcare technology providers and system developers, and patients.

In terms of evaluation, research can ultimately build on the concept map by developing generic measures for the concepts discussed. Currently, different theoretical frameworks inform the development of evaluation measures, which harms the possibility to make comparative evaluations of healthcare services, studies, initiatives, and policies [33]. Without comparability, it is difficult to find the most effective and efficient implementation strategies and solutions, which are in turn needed to prepare the way for a scientifically guided transfer of theory into healthcare services practice [34]. For example, Mc Allister et al. [33] consider patient empowerment as a patient-reported outcome measure (PROM) for healthcare services. However, they also conclude that a generic theoretical construct of the concept is needed first. When combining the need for a generic measurement instrument with the hypothesis that patient engagement is the most conclusive and furthest developed concept, the research question that must be answered is: How can a valid, feasible and reliable evaluation tool for patient engagement be created?

### Limitations

Reflecting on the methodology to create the concept map, a limitation of this work is subjectivity. In the systematic literature review, the involvement of two independent researchers during inclusion and exclusion criteria formulation and screening helped to ensure a neutral stance. The inconsistent usage of terms in prior literature is also a limitation of this work. The studies included in creating the concept map sometimes failed to incorporate alternative terms. As terms are being used so inconsistently, it would be essential to check if related terms might be used as synonyms. As multiple articles were generally used to justify the links between concepts, this effect was moderated. Furthermore, only considering the concepts in relation to personal healthcare could be a limitation of this paper, as attributes may vary or be added when also considering the meso- and macro-levels of healthcare (i.e., at an institutional level or in healthcare research and policymaking). Considering other levels of care, either separately or with respect to the findings in this paper, would be another task for future research.

A strength of this paper, especially in the context of existing research [5, 7, 12], is the breadth of concepts that were put in relation to one another. Instead of conceptualising a particular term, the entire subject area was covered to give an extensive overview of the various research streams that are dealing with concepts in the domain of patient engagement.

## Conclusions

In literature, the inconsistent and imprecise usage of terms to describe the active behaviour of patients in their healthcare leads to multiple problems in research, such as poorer communication and understanding, a lack of standard conceptual measures, and a deficiency in theory building and testing. These shortcomings ultimately lead to an impeded diffusion of theory into the practice of healthcare services. By creating a concept map, differences and relations of standard terms associated with patient engagement and patient empowerment were detected and systematically processed. The informational content needed to create the concept map was acquired using a systematic literature review.

The results achieved in a systematic, rigorous, and reliable manner contribute to a clearer understanding of terms and concepts related to patients having or taking on an active role in healthcare services. The added value of this work is for both researchers and interest groups from practice, such as healthcare system developers, policymakers, healthcare providers, and patients, who can use the results of our work as a steppingstone to impart momentum for successful development and implementation of patient engagement, patient empowerment, and associated approaches.

## Supplementary Information

**Additional file 1.** Final set of 17 articles derived from the systematic literature review.

**Additional file 2. **Contradicting statements found in the systematic literature review. 

## Data Availability

All data generated or analysed during this study are included in this published article [and its supplementary information files].
