# Supplementary material for: All together now – patient engagement, patient empowerment, and associated terms in personal healthcare
Source: BMC Health Serv Res. 2022 Sep 2;22:1116. doi: 10.1186/s12913-022-08501-5 (PMC9440506; doi:10.1186/s12913-022-08501-5)
Supplement: Supplementary file 1 — Additional file 1. Final set of 17 articles derived from the systematic literature review. [file 12913_2022_8501_MOESM1_ESM.pdf]

Additional file 1: Final set of 17 articles derived from the systematic literature review

Reference	Origin	Addressed Concepts	Methodology
Ahmed and Aslani 2014	Netherlands	Patient Adherence	Literature review
Bravo et al. 2015	United Kingdom	Patient Empowerment	Qualitative interviews, Scoping literature review
Castro et al. 2016	Belgium	Patient Empowerment, Patient Participation, Patient-Centeredness	Concept analysis based on the method by Avant and Walker (2005), Concept analysis of Haase et al. (1992)
Cerezo et al. 2016	Spain	Patient Empowerment	Literature review
Couture et al. 2018	Canada	Health Literacy, Patient Activation	Cross-sectional study of patients frequently using healthcare services
Deniz et al. 2021	Turkey	Shared decision-making, Compliance	Online questionnaire with participants that received a health service in the last year
Fumagalli et al. 2015	United Kingdom	Patient Empowerment, Patient Involvement, Patient Activation, Patient Engagement, Patient Enablement, Patient Participation	Literature review
Greene and Hibbard 2012	USA	Patient Activation, Patient Engagement	Cross-sectional study of patients in 35 primary care clinics
Higgins et al. 2017	USA	Patient Engagement	Concept analysis based on Rogers Evolutionary Method (2000)
Holmström and Röing 2010	Sweden	Patient Empowerment, Patient-Centeredness	Concept analysis based on the method by Avant and Walker (2005)

Kumar and Chattu 2018	India, Trinidad, Tobago	Patient-Centeredness, Person-Centeredness, Patient-directed care	Literature review
Menichetti et al. 2016	Italy	Patient Engagement, Patient Activation, Patient Empowerment, Patient Involvement, Patient Participation, Patient Adherence, Patient Compliance	Correspondence analysis, Literature review
Muscat et al. 2021	Australia	Health Literacy, Shared decision-making	Literature review
Nilsson et al. 2019	Finland, Sweden	Patient Participation	Concept analysis based on the method by Avant and Walker (2013)
Robinson et al. 2008	USA	Patient-Centeredness, Adherence	Literature review
Sahlsten et al. 2008	Sweden	Patient Participation	Concept analysis based on the method by Avant and Walker (1995)
Scholl et al. 2014	Germany	Patient-Centeredness	Literature review
