# Supplementary material for: All together now – patient engagement, patient empowerment, and associated terms in personal healthcare
Source: BMC Health Serv Res. 2022 Sep 2;22:1116. doi: 10.1186/s12913-022-08501-5 (PMC9440506; doi:10.1186/s12913-022-08501-5)
Supplement: Supplementary file 2 — Additional file 2. Contradicting statements found in the systematic literature review. [file 12913_2022_8501_MOESM2_ESM.pdf]

Additional file 2: Contradicting statements found in the systematic literature review

<table>
<tr><th>Literature finding 1</th><th>Literature finding 2</th><th>Decision for the concept map</th></tr>
<tr><td colspan="3">Patient engagement</td></tr>
<tr><td>The patient has the ability to engage in self-care.
(Higgins et al. 2017)</td><td>The patient only has the willingness to engage, not necessarily the ability.
(Fumagalli et al. 2015)</td><td>In accordance with finding 1:
Patient engagement means the patient has the ability (not only the willingness) to engage in self-care.</td></tr>
<tr><td colspan="3">**Reasoning:** Patient empowerment and patient activation, meaning a patient’s gain of skills and confidence, are a precondition to patient engagement. Expressed the other way around, patients can only engage in care if they are empowered. This reasoning is backed by several different sources, including Menichetti et al. (2014), Cerezo et al. (2016), and Holmström and Röing (2010).</td></tr>
<tr><td colspan="3">Patient involvement and patient participation</td></tr>
<tr><td>Both concepts focus on the patient exclusively rather than the relationship and interaction with the health care provider.
(Higgings et al. 2017)</td><td>In contrast to patient involvement, patient participation is dependent on the existence of a relationship with the healthcare provider.
(Nillsson et al. 2019, Sahlsten et al. 2008, Fumagalli et al. 2015)</td><td>In accordance with finding 2:
The concepts of patient involvement and patient participation differ when considering the necessity for a relationship with the provider.</td></tr>
<tr><td colspan="3">**Reasoning:** More sources agree that patient participation occurs in relation to a health care provider. Also, the focus of the studies was decisive. Sahlsten et al. (2008) and Nillsson et al. (2019), in contrast to the other papers, concentrate directly on the concept of patient participation and therefore, arguably, have a deeper insight into this concept.</td></tr>
<tr><td colspan="3">Patient activation and health literacy (patient enablement)</td></tr>
<tr><td>There is no association between the two concepts for frequent users of health services with a chronic disease, but it could be the case for other patient types.
(Couture et al. 2018)</td><td>Patients that understand their health condition and feel able to cope with it (enabled patients) can participate in self-care.
(Fumagalli et al. 2015)</td><td>In accordance with finding 2:
Enabled patients have the ability for self-care, implying that patient enablement can lead to patient involvement. For patient involvement to occur, a patient must be empowered/ activated.</td></tr>
<tr><td colspan="3">**Reasoning:** In a primary study, Couture et al. (2018) investigate if patients’ health literacy influences patients’ activation. No significant association was found in a population of frequent users of health services with chronic conditions. However, Couture et al. (2018) also report five studies that found a positive association between health literacy and patient activation. It is stated that these studies often included samples of patients with higher activation or health literacy levels compared to the general population. Therefore, they</td></tr>
</table>

conclude that there may be an association between the concepts, but only for specific (but yet undefined) populations. In contrast, Fumagalli et al. (2015) performed a systematic literature review and reasoned that enablement could lead to patient involvement.

It must be considered that the study settings differ immensely, the first being a quantitative and the second a qualitative study. However, they only contradict each other to a limited extent. First, Fumagalli et al. (2015) only state that enablement *can* lead to involvement. Second, Couture et al. (2018) does not find a correlation for a specific population, noting that other primary studies have found correlations when focusing on different population groups. When generally considering what is needed for patients to get involved in their healthcare, the proliferation of their healthcare knowledge and skills will certainly play a role, even if this has not been proven for all population groups in primary studies. For concept map creation, it was assumed that patient enablement (incorporating health literacy) leads to a patient being empowered. However, as explained in our work, there are conditions preventing one concept from leading to another. For example, a patient could have a high health literacy and consciously choose not to become more active in their healthcare.
